# The “Melancholy Days”*Reproduced from an early issue of the “Red Back,” when first ye editor’s harp was strung, or when first he “struck his tuneful lyre.”

**Published:** 1901-11

**Authors:** 


### THE "MELANCHOLY DAYS."*

"The melancholy days are come," (says Bryant,
But he lived in Connecticut,) "the saddest
Of the year"; but just where the sad comes in
I am, just now, not able to perceive;—
Can't see it.

With its balmy air and zephyr-breezes,—
("Wailing winds and naked trees" nothing),
The sense of hush,—the gorgeous trees-es—
The purple mist that dims the distance—
October is just splendid!

Why, it's harvest time! See the pumpkins!
And the peas-es! and the pinders! and perhaps—
Potatoes (or po-turnips), how they tumble in to market!
And the farmer? Is he happy? Rather!
Where's the melancholy?

Purple are the grapes-es now, and juicy
Are the apples. Nuts are brown. In the forest
Sings the mocker, (thinks it's spring;)
Red the roses in the garden; and subscriptions
Are just *pouring* in;—where's the sad?

*Reproduced from an early issue of the "Red Back," when first ye editor's harp was strung, or when first he "struck his tuneful lyre."

See yon ripple on the water! (imagine the water;)
  Why's its solemn stillness thusly broken?
I guess a big old trout has taken
  In a fly;—I'll take *him* in!
        (Look! beauty, ain't he?)

And now the doctor hies him outward; now's the time
  He's got to rustle; (not to see a patient
But to get his money;) and with Christian charity
  Forthwith *he* remembers the poor——
        Editor; (please remit, doctor!)

Heaped in the hollow of his desk the doctor's bills are laid,
  He'll rustle 'mongst his patrons till the last darned one is paid.
The farmer and his men are flush, and in their eyes he sees—
  Speculation? No! but *cash*—to pay—in lieu of —*peas!*†
        Oh, where's the melancholy?

[†For fear that this beautiful point may not be clearly seen by the city physician, it is necessary to explain that too often the poor, tired country doctor has to take his "pay in kind"—in "chips and whetstones"—farm products, or anything he can get. One rustling doctor—a friend of ours—wrote us that he "collected on account" (in one day) a parrot, two canary birds, four bushels of black-eye peas, and a calf worth four dollars! Lucky doctor;—"and," he added, "the farmer 'throwed in' a cat!" Fact!]

